# Artificial Intelligence and Digital Tools Across the Hepato-Pancreato-Biliary Surgical Pathway: A Systematic Review

**DOI:** 10.3390/jcm14186501

**Published:** 2025-09-15

**Authors:** Andreas Efstathiou, Evgenia Charitaki, Charikleia Triantopoulou, Spiros Delis

**Affiliations:** 1 Department of Surgery, Konstantopouleio General Hospital, 14233 Athens, Greece; jenny_charitaki@hotmail.com (E.C.); spirosgdelis@gmail.com (S.D.); 2 Department of Radiology, Konstantopouleio General Hospital, 14233 Athens, Greece; ctriantopoulou@gmail.com

**Keywords:** hepatopancreatobiliary surgery, artificial intelligence, augmented reality, image-guided surgery, robotic surgery, machine learning, surgical planning, intraoperative navigation, predictive analytics, deep learning, systematic review

## Abstract

**Background:** Hepato-pancreato-biliary (HPB) surgery involves operations that depend heavily on precise imaging, careful planning, and intraoperative decision-making. The rapid emergence of artificial intelligence (AI) and digital tools has assisted in these domains. **Methods:** We performed a PRISMA-guided systematic review (searches through June 2025) of AI/digital technologies applied to HPB surgical care, including novel models such as machine learning, deep learning, radiomics, augmented/mixed reality, and computer vision. Our focus was for eligible studies to address imaging interpretation, preoperative planning, intraoperative guidance, or outcome prediction. **Results:** In total, 38 studies met inclusion criteria. Imaging models constructed with AI showed high diagnostic performance for lesion detection and classification (commonly AUC ~0.80–0.98). Moreover, risk models using machine learning frequently exceeded traditional scores for predicting postoperative complications (e.g., pancreatic fistula). AI-assisted three-dimensional visual reconstructions enhanced anatomical understanding for preoperative planning, while augmented and mixed-reality systems enabled real-time intraoperative navigation in pilot series. Computer-vision systems recognized critical intraoperative landmarks (e.g., critical view of safety) and detected hazards such as bleeding in near real time. Most of the studies included were retrospective, single-center, or feasibility designs, with limited external validation. **Conclusions:** The usage of AI and digital tools show promising results across the HPB pathway—from preoperative diagnostics to intraoperative safety and guidance. The evidence to date supports technical feasibility and suggests clinical benefit, but routine adoption and further conclusions should await prospective, multicenter validation and consistent reporting. With continued refinement, multidisciplinary collaboration, appropriate cost effectiveness, and attention to ethics and implementation, these technologies could improve the precision, safety, and outcomes of HPB surgery.

## 1. Introduction

Hepato-pancreato-biliary (HPB) diseases such as hepatocellular carcinoma, pancreatic ductal adenocarcinoma, cholangiocarcinoma, and complex benign conditions often require high-stakes surgical intervention. Surgical resection remains the cornerstone of curative treatment for many of these diseases, but HPB operations are among the most challenging in abdominal surgery due to intricate anatomy, critical vasculature, and narrow margins for error. Even in experienced centers, outcomes like negative resection margins, complication rates, and long-term survival are closely tied to the quality of preoperative imaging, the surgeon’s planning, and real-time intraoperative judgment [1]. In recent years, HPB surgery has also trended toward minimally invasive approaches (laparoscopic and robotic surgery) to improve recovery, although these techniques demand even greater precision and visualization given the reduced tactile feedback and view [2].

Artificial intelligence (AI) has rapidly advanced in medicine and offers powerful tools to address some of these needs. AI—particularly machine learning (ML) and deep learning algorithms—excels at detecting patterns in complex data, often surpassing human performance in tasks like image recognition. In the domain of medical imaging, AI applications have grown exponentially; a recent bibliometric analysis identified 2552 publications on AI in HPB surgery from 2014 to 2024, with “diagnosis” and “CT” (computed tomography) among the most frequent keywords [3]. This reflects a contemporary research focus on using AI for disease detection and characterization in imaging, especially for liver and pancreatic tumors. Indeed, AI-driven image analysis can potentially identify subtle features on radiologic scans or intraoperative ultrasound that might be missed by the human eye or predict tumor behavior (such as malignancy or likelihood of recurrence) from quantitative patterns (“radiomics”). Beyond preoperative imaging, AI techniques are being explored for patient risk stratification (e.g., predicting postoperative complications), operative planning (e.g., automated 3D reconstructions and virtual simulations), and intraoperative guidance (e.g., augmented reality overlays and real-time video analysis) [4]. The breadth of “digital tools” thus encompasses not only AI/ML algorithms but also computer-vision, sensor fusion, and enhanced reality systems—all aiming to support surgical decision-making from start to finish [5,6]. Recent cross-domain evidence underscores these advances in diagnostics and imaging [7,8,9].

Early results specific to HPB surgery are encouraging. For instance, multiple studies have shown that ML models can predict postoperative pancreatic fistula more accurately than traditional surgeon-calculated scores [10,11]. On the imaging front, convolutional neural networks (CNNs) have been trained to detect liver tumors or differentiate pancreatic lesions on scans with high sensitivity—in some cases identifying tumors months earlier than standard diagnostics [12,13]. In the operating room, augmented reality (AR) systems have been piloted to project virtual anatomy onto the surgical field to help surgeons navigate internal structures [14,15,16]. Computer-vision algorithms can process laparoscopic video in real time, for example, by recognizing unsafe tissue planes or early signs of bleeding and alerting the team, allowing proactive intervention [17]. These developments align with the broader trend of “smart surgery,” wherein digital technology acts as a co-pilot to the surgeon. Beyond malignancy, benign biliary disease such as hepatolithiasis poses complex anatomic and management challenges. Contemporary Western case-series data underscore the role of advanced imaging (e.g., MRCP and cholangioscopy) in delineating ductal pathology and guiding treatment, reinforcing the need for high-fidelity 3D planning even outside oncologic indications [18].

Despite growing interest and a proliferation of pilot studies, there remains a gap before these innovations are widely adopted in clinical practice. Most AI tools in HPB surgery are still in the developmental or experimental stage, tested on retrospective datasets or small case series. Validation in prospective clinical trials is needed for regulatory approval and to build clinical confidence. Surgeons must also be able to trust and seamlessly interact with these systems during real operations. Additionally, issues of integration (how to incorporate AI outputs into surgical workflows), regulation, and ethics (such as liability and transparency of AI decision-making) need to be addressed. Given the rapid pace of new data, a timely systematic review is warranted to synthesize the current evidence, evaluate study quality, and guide clinicians and researchers on future directions of AI in HPB surgery. Herein, we present a comprehensive systematic review of AI and digital tools in HPB surgery, adhering to PRISMA 2020 guidelines, and discuss the clinical advances and challenges in this emerging field.

The aim of this study is to synthesize and appraise HPB relevant AI digital tools used across the perioperative spectrum (diagnostics, planning, intraoperative guidance, and predictive analysis), focusing on performance, quality, and readiness for integration in clinical everyday practice.

This review advances the prior literature by providing an end-to-end synthesis across the entire HPB surgical pathway (preoperative imaging/planning, intraoperative AR/MR and computer-vision guidance, postoperative risk prediction) updated through June 2025; we (i) include intraoperative CV/MR alongside imaging and predictive analytics; (ii) apply study-appropriate appraisal (PROBAST, QUADAS-2) with quantitative bias/validation summaries; (iii) map evidence by study design and readiness; and (iv) translate findings into practical implementation guidance.

In contrast to prior HPB AI surveys or AR-only reviews, we excluded purely radiology-only studies without surgical relevance, emphasized external validation/readiness, and integrated ethics/implementation aspects specific to surgical workflows.

## 2. Materials and Methods

### 2.1. Protocol and Registration

This systematic review was performed in accordance with the PRISMA 2020 guidelines. The review protocol (objectives, search strategy, inclusion criteria, and analysis methods) was formulated prior to commencing study selection. The protocol was not registered in PROSPERO or other public databases given the rapid update cycle and heterogeneous technology scope; however, all methods followed PRISMA recommendations for transparent reporting [19]. There were no amendments to the initial protocol after commencement of the review. The full PRISMA 2020 checklist is available in Supplementary Table S1.

### 2.2. Eligibility Criteria

Inclusion: English-language human studies evaluating AI/digital tools (ML/DL, radiomics, AR/MR, computer vision, navigation/3D planning) within an HPB surgical context—imaging interpretation, preoperative planning/simulation, intraoperative guidance, or outcome prediction. Any empirical design (retrospective/prospective observational, feasibility/pilot, RCT).

Exclusion: conference abstracts without full data; editorials/commentaries; animal/phantom-only work without clinical translation; purely diagnostic radiology studies with no surgical context.

### 2.3. Information Sources and Search Strategy

A comprehensive literature search was conducted in four databases: PubMed, Embase, Web of Science, and Scopus. We also searched engineering and specialty databases—IEEE Xplore and ACM Digital Library—for relevant technical papers on surgical navigation or AR that might not be indexed in the medical databases. Additionally, the Cochrane Library was searched for any pertinent systematic reviews. The search was first performed in May 2025 and updated in June 2025 to capture newly published studies.

The search strategy combined keywords and controlled vocabulary terms related to two domains: (1) HPB surgery (e.g., “hepato-pancreato-biliary” OR “HPB” OR “liver surgery” OR “hepatectomy” OR “pancreatic surgery” OR “Whipple” OR “bile duct surgery”, etc.) and (2) Artificial Intelligence/Digital Tools (e.g., “artificial intelligence” OR “machine learning” OR “deep learning” OR “neural network” OR “radiomics” OR “augmented reality” OR “mixed reality” OR “surgical navigation” OR “image-guided surgery” OR “3D reconstruction” OR “robotic surgery” OR “decision support” OR “predictive model”, etc.).

These were combined with Boolean operators AND/OR. A sample PubMed query was:

((“hepato-pancreato-biliary” OR HPB OR hepatobiliary OR pancreatic OR pancreatoduodenectomy OR hepatectomy OR “liver resection” OR “pancreatic resection”)

AND

(“artificial intelligence” OR “machine learning” OR “deep learning” OR radiomics OR “augmented reality” OR “mixed reality” OR “image-guided” OR navigation OR “3D planning” OR “3D reconstruction” OR robotic OR “decision support” OR “predictive model”))

AND 2018:2023[dp] AND english[lang].

Similar queries were adapted for the other databases. We also hand-searched reference lists of relevant articles and conference proceedings to ensure completeness. No date restrictions were imposed initially, but practically most retrieved studies were from the last decade given the nascent nature of AI in surgery.

### 2.4. Study Selection

**Figure 1:**
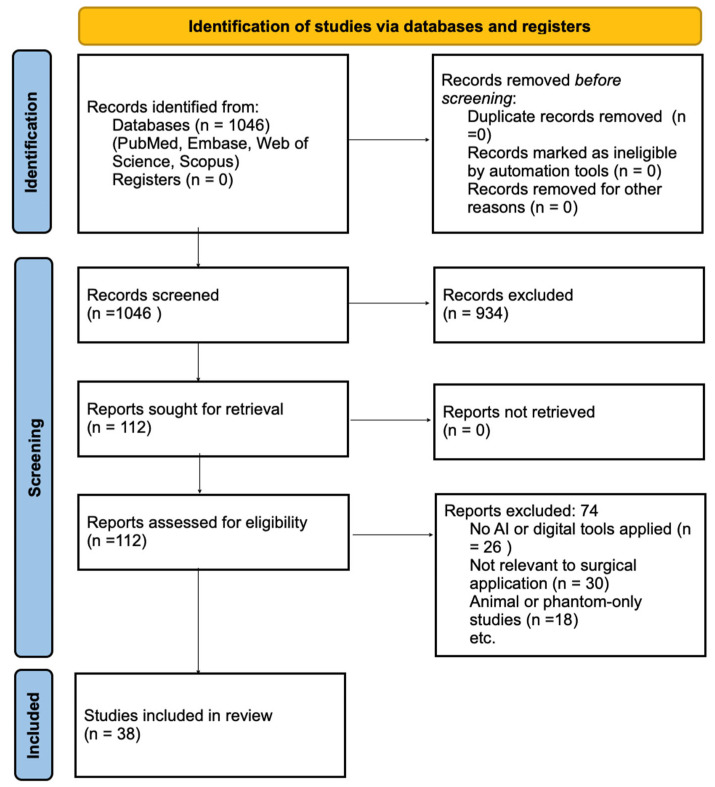
A PRISMA 2020 flow diagram details the number of records identified, screened, excluded, and finally included.

All identified records were imported into a reference manager, and duplicates were removed. Two reviewers independently screened titles and abstracts for relevance. Studies deemed potentially eligible or unclear were retrieved in full text. The same two reviewers then independently evaluated full-text articles against the inclusion criteria. Any disagreements were resolved by discussion and consensus, involving a third reviewer if needed. A PRISMA 2020 flow diagram (Figure 1) details the number of records identified, screened, excluded, and finally included. The most common reasons for exclusion at full text were absence of any AI/digital tool specific to HPB surgery (e.g., purely diagnostic radiology studies without surgical context), non-clinical studies (e.g., algorithm papers with no clinical validation), or outcomes not relevant to surgical planning or outcomes.

### 2.5. Data Extraction

Two reviewers independently extracted data using a standardized form. From each included study, we collected the author, year, study design (retrospective, prospective, etc.), sample size and population (e.g., number of patients or images, disease focus like HCC or PDAC), type of AI or digital tool (including details of algorithms or platforms), the application domain (imaging diagnosis, risk prediction, planning, intraoperative guidance, etc.), and key outcomes or findings. For prediction model studies, we noted performance metrics like AUC, sensitivity, specificity, etc. For AR/navigation tools, we noted qualitative and quantitative outcomes such as registration error, time saved, or user feedback on usefulness. Any additional notable findings (e.g., technical challenges) were also recorded. The two extractors compared their results and resolved discrepancies by consensus.

### 2.6. Risk of Bias Assessment

We assessed methodological quality and risk of bias using appropriate tools for each study design. For prediction model studies (e.g., algorithms to predict postoperative complications or recurrence), we used the Prediction Model Risk of Bias Assessment Tool (PROBAST) [20]. This evaluates bias across four domains (participants, predictors, outcome, analysis), rating each as a low, high, or unclear risk of bias. For diagnostic accuracy studies of AI in imaging, we applied criteria based on QUADAS-2 to judge bias in patient selection, index test, reference standard, and flow/timing [21]. For exploratory/feasibility studies of intraoperative tools where formal checklists didn’t directly apply, we qualitatively considered factors such as highly selective samples or lack of appropriate comparator. Two reviewers performed the quality assessments independently, and disagreements were resolved by discussion, and when necessary, a third reviewer provided a final decision. We incorporated risk-of-bias findings into our interpretation of results, giving more weight to findings from higher-quality studies. Overall, many included studies had at least some risk of bias (often due to retrospective designs or single-center data); these limitations are noted in our discussion.

### 2.7. Data Synthesis

Given heterogeneity of interventions and outcome metrics, a meta-analysis was not feasible. We therefore conducted a narrative synthesis with descriptive subgroup analyses by major application domains of AI/ digital tools in HPB surgery. We grouped results into categories corresponding to stages of surgical care:

1. Preoperative Imaging and Diagnosis
2. Risk Prediction (outcomes)
3. Surgical Planning and Simulation
4. Intraoperative Guidance and Navigation
5. Surgical Video Analysis/Robotics

Within each category, we summarize the performance of AI tools and any reported impact on clinical decision-making or patient outcomes. Where appropriate, we compare AI tool performance to conventional standards (e.g., AI vs. human radiologist, or vs. existing risk scores). All analyses are descriptive; no pooled summary measures were calculated due to diverse data. Instead, we highlight trends, such as areas where multiple studies consistently show benefit versus areas of conflicting or limited evidence.

## 3. Results

### 3.1. Study Selection and Characteristics

**Figure 2:**
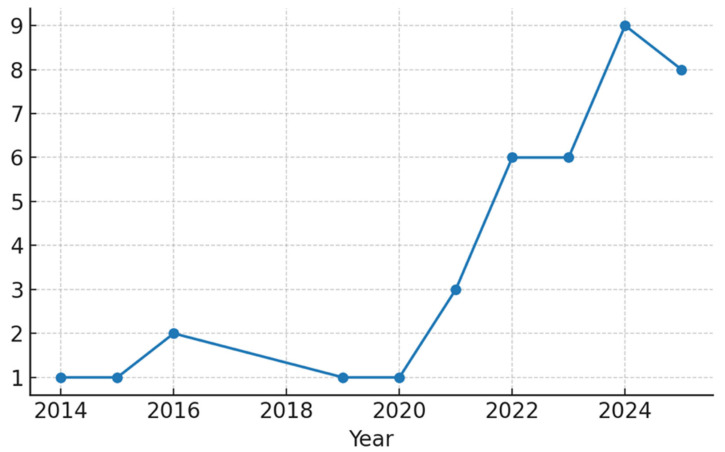
Publication trends (included studies per year).

**Figure 3:**
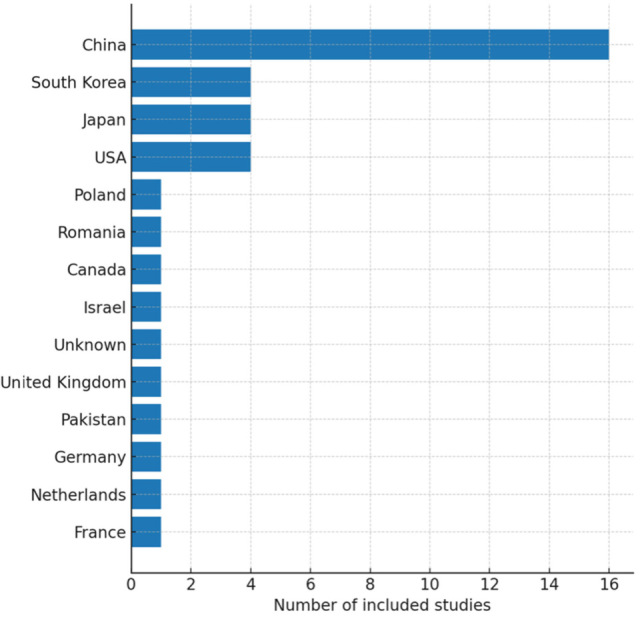
Geographic distribution (by first-author country).

**Figure 4:**
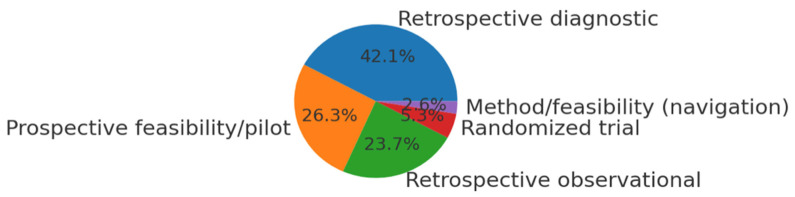
Study design distribution.

The database search yielded 1046 unique records. After title/abstract screening, 112 articles remained for full-text review. Of these, 38 studies met our inclusion criteria and were included in the qualitative synthesis. The PRISMA flow diagram (Figure 1) illustrates the study selection process and reasons for exclusion. Publication activity has steadily increased over the past decade, with a marked rise after 2020 (Figure 2). The included studies originated from a wide range of countries, with China, Japan, the USA, and European nations contributing the majority (Figure 3). Regarding study design, most were retrospective diagnostic or observational series, with fewer prospective feasibility studies and only two randomized controlled trials (Figure 4).

No clear indication of reporting bias was identified qualitatively; however, potential publication bias favoring positive outcomes could not be entirely ruled out. Moreover, the certainty of evidence across included studies was moderate to low due to predominantly retrospective designs, lack of external validation, and small sample sizes, limiting the generalizability of findings. We also tabulated key characteristics of representative primary studies from our set (Table 1). Table 1 presents representative primary studies organized by surgical pathway and application domain; formal study-design classification and counts are reported in Figure 4, with per-study details in Supplementary Table S2.

### 3.2. Preoperative AI and 3D Planning (Imaging and Diagnosis)

Several studies applied AI to interpret preoperative imaging for HPB diseases—such as ultrasound, computed tomography (CT), and MRI—with the aim of improving diagnostic accuracy.

- Machine learning for tumor detection and characterization: In liver imaging, AI algorithms (often CNNs) have shown high accuracy in identifying liver tumors and classifying lesion types. For example, one study combined radiomics features with a CNN to differentiate hepatocellular carcinoma (HCC) from liver metastases on ultrasound images, achieving AUC ~0.85–0.90 [4]. In another, a deep learning model (ResNet-50) trained on endoscopic ultrasound (EUS) images distinguished pancreatic ductal adenocarcinoma (PDAC) from chronic pancreatitis with an AUC of 0.95 [22]. These accuracies are on par with, or exceeding, expert radiologist performance in these tasks. A recent systematic review of AI in liver imaging noted that most algorithms focus on lesion classification (particularly on CT scans) and report high internal accuracies, but a common limitation is lack of external validation [28]—suggesting potential optimism bias in those results.
- Beyond simply detecting lesions, AI has been used to predict tumor biology or staging from preoperative scans. For instance, radiomic models analyzing preoperative CT have shown ability to predict early HCC recurrence after resection better than conventional clinical staging, by identifying subtle textural features associated with tumor aggressiveness [30]. In pancreatic cancer, one model automatically quantified vascular involvement on CT to classify tumors as resectable vs. locally advanced unresectable, with high agreement to expert assessments [31]. Tools like this could aid surgical decision-making by more objectively staging tumors preoperatively (e.g., determining which patients should go straight to surgery vs. need neoadjuvant therapy).
- Some digital tools also bridge into intraoperative imaging enhancement. For example, an experimental system used an AI algorithm to register real-time intraoperative ultrasound with preoperative CT images [32]. This kind of image fusion could improve the surgeon’s ability to locate tumors during laparoscopic ultrasound by correlating it with preop imaging. In summary, AI in preoperative imaging has shown high diagnostic performance in identifying and characterizing HPB tumors. The challenge ahead lies in translating these algorithms into clinical workflows and ensuring they maintain accuracy in the real-world setting. As noted in the literature, prospective validation is needed: recent study concluded that while ultrasound-based AI models showed AUCs up to 0.98 in differentiating tissue types, prospective studies are required to confirm consistent performance externally [4].
- In parallel, advanced 3D imaging and visualization tools are augmenting preoperative planning. Automated 3D reconstruction: Several studies highlighted the use of AI to perform 3D reconstructions of liver and pancreatic anatomy from CT/MRI scans. This includes segmenting the liver into anatomical units and delineating tumors, vessels, and ducts [33,34]. Historically, creating patient-specific 3D models was time-consuming (often requiring hours of manual work by radiologists). AI-based image segmentation significantly accelerates this. For example, a deep learning algorithm in one study reduced liver 3D model processing time by ~94% compared to manual methods. Rapid model generation makes it feasible to use 3D visualization for every complex case. Surgeons can then virtually plan their resections—determining optimal resection planes, estimating future liver remnant volume, and even rehearsing the surgery on a computer [35,36]. One recent randomized study even incorporated 3D-printed models: it was reported that using AI-assisted 3D printed liver models for surgical planning led to significantly less intraoperative blood loss compared to standard planning without such models. This suggests that enhanced planning can translate to improved intraoperative outcomes [37].
- Augmented and mixed reality for planning: In addition to screen-based 3D models, some teams have explored using augmented reality (AR) or mixed reality to aid preoperative planning. For instance, holographic visualization of patient anatomy via AR headsets has been tested as a planning tool. In a pilot study for pancreatic tumor resection using mixed reality, surgeons could visualize major arteries and veins holographically in 3D prior to surgery, which reportedly improved their confidence in dissection around those structures (though quantitative outcomes were not yet measured) [38,39].

These technologies blur the line between preoperative planning and intraoperative guidance, as the same AR models can potentially be brought into the OR (see below).

### 3.3. Predictive Analytics (AI for Risk and Outcome Prediction)

Another major application of AI in HPB surgery is prediction of operative risk and postoperative outcomes. These AI-driven predictive analytics models use preoperative or intraoperative data to forecast complications and aid in risk stratification and clinical decision-making.

- Postoperative complication risk models: A prototypical example is predicting postoperative pancreatic fistula (POPF), one of the most common and feared complications after pancreatic resection. Traditional risk scores (like the Fistula Risk Score, FRS) use a few surgeon-assessed variables and have moderate accuracy (AUC often ~0.6) [40]. Several included studies developed ML models that substantially improved prediction of clinically relevant POPF. For instance, in a recent study, an ensemble of ML algorithms was applied to ~300 pancreaticoduodenectomies; their model incorporated dozens of clinical and radiologic features and achieved an AUC around 0.80 for POPF on validation, significantly outperforming the conventional FRS (~0.56 AUC) [24]. This highlights AI’s value in personalized risk stratification by analyzing complex, high-dimensional perioperative data that surgeons cannot easily synthesize alone. Similarly, other studies used ML to predict complications like liver insufficiency after hepatectomy or severe bile leak after bile duct injury repair, often reporting improved accuracy over logistic regression models [41,42]. Notably, most AI-based risk models to date have been developed retrospectively; prospective testing is still scarce. A consistent finding, however, is that combining human expertise with AI can be synergistic—e.g., an AI model might quickly flag high-risk patients, which the surgical team can then further evaluate and proactively manage (such as closer postoperative monitoring or preemptive interventions) [43,44].
- Outcome prediction and decision support: Beyond immediate complications, AI has been used to predict oncologic outcomes like cancer recurrence or long-term survival. Models integrating tumor genomic data, radiologic features, and patient comorbidities have shown promise in forecasting which patients are likely to have early recurrence after resection, which could influence adjuvant therapy decisions. For example, one radiomics study could predict early recurrence of HCC from preoperative imaging and lab data, raising the prospect of tailoring follow-up intensity for high-risk patients [45]. Another AI tool predicted which patients might not benefit from surgery at all due to very aggressive tumor biology, potentially guiding multidisciplinary discussions on alternative treatments [46]. These predictive analytics tools carry ethical implications: using an AI to recommend against surgery or to alter standard treatment must be approached cautiously and transparently. Nonetheless, they offer an avenue for more data-driven clinical decisions in HPB oncology.

In summary, AI-based predictive models in HPB surgery show clear potential to enhance risk assessment, but their clinical integration is still in early phases. Surgeons could use such models to better inform patients of risks, optimize perioperative planning (e.g., allocate ICU beds for high-risk cases), or select patients for certain interventions. Importantly, any AI risk estimate must be interpreted in context—these tools should assist, not replace, clinician judgment. Most models need prospective validation; as one study pointed out, it will be critical to test AI risk predictions in real-time and see if acting on them actually improves patient outcomes. Moreover, issues of bias and generalizability exist: many models were trained on single-institution data and may not perform as well elsewhere (some risk factors might be center-specific). The push for external validation and eventual randomized trials (e.g., using AI risk tools versus standard care) is ongoing [47].

### 3.4. Intraoperative AR and Navigation

One of the most visually compelling applications of these technologies is augmented reality (AR) for intraoperative guidance. AR in surgery involves overlaying digital information (such as anatomical models, tumor locations, or navigation markers) onto the surgeon’s field of view in real time, effectively providing a “surgical GPS.” This can be implemented via specialized goggles or head-mounted displays (like the Microsoft HoloLens), or by overlaying on laparoscopic/robotic camera feeds on a monitor [26].

- AR for open and minimally invasive surgery: Several pilot studies in our review implemented AR during liver or pancreatic resections. The common workflow is as follows: a 3D model of the patient’s anatomy (from preoperative imaging) is created and then registered to the patient on the operating table. Registration often uses surface landmarks or fiducial markers so that the virtual model aligns correctly with the real organ. For example, in an AR-guided liver surgery study, a CT-based 3D model of the liver (with tumor and vessels) was projected onto the live laparoscopic camera view [48]. For instance, a snapshot of such an AR overlay is being shown to the surgeon for a planned segment 5 liver resection, with the tumor (green) and planned transection plane (red) delineated. By seeing this overlay, the surgeon obtains a form of “X-ray vision”—the ability to visualize hidden structures beneath the liver surface. This can guide where to cut and help avoid critical vessels that are not visible externally [26,49].
- Technical feasibility and accuracy: The feasibility of AR has been demonstrated in small case series. A recent study reported using an AR overlay in three cases of robotic liver resection. The AR model was displayed in the surgeon’s console view, showing tumor and vasculature projections during the robotic hepatectomies. Surgeons found it helped localize lesions and plan transections. However, a noted limitation was the need for improved registration accuracy—misalignment of the virtual overlay by even a few millimeters could erode trust in the system. Organ deformation is a major issue: in soft tissues like the liver, once surgery begins and the organ is mobilized or resected partially, it changes shape, making static preoperative overlays increasingly inaccurate [26]. Future solutions may involve real-time organ tracking or deformable models (potentially using AI to adjust the overlays continuously based on intraoperative imaging or sensors) [50,51].
- Navigation and workflow: Despite these challenges, AR clearly adds value as a navigation aid. For example, AR has been used to guide the placement of trocars (ports) in minimally invasive HPB surgery by projecting an optimal port map onto the patient’s abdomen [26,52]. It has also been applied to help locate small tumors during parenchymal transection by projecting their approximate depth/location [52]. Surgeons generally still rely on tactile feedback or intraoperative ultrasound for confirmation, but AR provides an extra layer of information. In a recent mixed-reality study for pancreatic tumor resection, surgeons could see major blood vessels holographically “through” the pancreatic tissue, which improved their confidence in dissecting around those structures (qualitatively reported) [53].

Current evidence for AR’s benefits is still low-level (case series and simulation studies), but almost all reports indicate it is technically achievable and subjectively improves the surgeon’s situational awareness. Quantitative outcome improvements (like reduced positive margin rates or operative time) have yet to be robustly demonstrated. There is also a learning curve and significant setup required (segmentation, calibration in the OR). A 2021 review noted that AR in HPB surgery is “far from standardized” due to high complexity and cost [23]. Nonetheless, rapid improvements in computing power and graphics are making real-time AR more viable. We recommend that high-volume HPB centers and device companies continue to refine AR navigation systems, focusing on improving registration accuracy and user ergonomics. Meanwhile, practicing surgeons should consider participating in trials of AR guidance, as these technologies could foreseeably reduce guesswork in the future—for example, ensuring a bile duct tumor margin is clear by seeing ducts highlighted, or avoiding critical arteries in a difficult resection by having them virtually outlined [54].

### 3.5. Robotics and AI Augmentation (Computer Vision in Surgery)

Another frontier is the use of AI-driven computer vision to analyze surgical video in real time, particularly in minimally invasive surgery. HPB procedures like laparoscopic cholecystectomy, liver resection, or pancreaticoduodenectomy generate rich video data from the endoscopic camera. By applying computer vision algorithms (usually deep learning models), this video stream can be analyzed to identify structures, surgical phases, or impending dangers, and then provide feedback or alerts to the operating surgeon [55,56,57].

- Automatic landmark and safety identification: A prominent example is objective recognition of the critical view of safety (CVS) during laparoscopic cholecystectomy. CVS is a method to ensure the cystic duct and artery are clearly identified before cutting, to prevent common bile duct injuries. Achieving CVS is typically a subjective assessment by the surgeon. One included study developed an AI system to automatically detect when CVS had been attained. They trained a deep CNN on ~23,000 laparoscopic cholecystectomy video frames, labeled for presence or absence of key CVS criteria. The best model achieved ~83% accuracy in determining if CVS was achieved, operating at ~6 frames per second (nearly real-time). Such a system could potentially alert a surgeon if they have not yet adequately exposed critical structures [23]. In effect, the AI serves as a “safety coach” in the OR. Considering bile duct injuries are among the most dreaded complications in HPB surgery, this application has high significance. A recent review echoed that AI can reliably identify safe vs. unsafe zones during gallbladder dissection, reinforcing its potential to improve surgical safety [58].

Beyond CVS, computer vision has been used to detect other anatomical landmarks in real time. Algorithms have been trained, for instance, to recognize the cystic duct, cystic artery, and common bile duct, or specific liver segments in the laparoscopic view. Early results are promising: a recent review reported that such AI systems can detect landmarks with reasonable sensitivity, though challenges remain when the view is obscured by bleeding or fat [59].

- Real-time hazard detection: Another cutting-edge application is automatic detection of intraoperative bleeding and other hazards. In complex HPB surgeries, bleeding from small vessels can sometimes go unnoticed for critical seconds if the surgeon’s focus is elsewhere. One innovative study designed a hazard detection system using the YOLOv5 deep learning model to identify bleeding in endoscopic video and immediately display alerts via an AR headset. The system distinguished true bleeding from look-alike events (such as spilled irrigating fluid) with high accuracy, highlighting the bleed with a bounding box on the surgeon’s display in real time. Essentially, this is an AI-driven early warning system—a digital “co-pilot” that never blinks, constantly scanning the operative field for hazards. In testing across recorded procedures, the system successfully alerted surgeons to bleeding events that were at times outside the central field of view or at the periphery. Such technology could reduce response time to hemorrhage, potentially improving patient safety in major liver or pancreatic resections where even a brief delay in controlling bleeding can have serious consequences [27].
- Robotic surgery integration: In robotic surgery, integration of AI is also advancing. The da Vinci surgical system already allows some digital interfacing—for example, the TileProTM feature can display auxiliary imaging (like preoperative scans or ultrasound) side-by-side with the operative view [60]. Researchers are going further by embedding AI into robotic workflows. One aspect is surgical skill analysis—computer vision can analyze the motion of instruments (captured via the robot’s kinematic data or video) to grade the surgeon’s skill or identify suboptimal technique. While our review focused on clinical outcome tools, it is worth noting that some surgery studies have used AI to assess technique quality for training purposes [61].

Perhaps the most futuristic concept is partially or fully autonomous robotic actions. In experimental settings outside HPB, AI has been used to automate certain surgical subtasks. For example, a research group demonstrated an autonomous laparoscopic intestinal anastomosis where an AI-guided robot performed suturing in an animal model [62]. Moreover, there is a report of an autonomous camera navigation system: an AI controlled the endoscope to keep the instruments and target in view at all times, which could impact minimally invasive HPB surgeries in the future. While these early autonomies do not directly improve patient outcomes yet, they reduce the need for an assistant and provide steadier visualization. In our review, no study reported a fully autonomous major surgical step in humans (nor would that be ethical at present), but these developments lay the groundwork. Experts envision that AI might handle routine parts of surgery (like optimally adjusting the camera or monitoring for bleeding) so that surgeons can focus on critical decisions and maneuvers [63].

### 3.6. Postoperative Video Analytics

A concrete benefit of these advances, already being realized, is in operative documentation and analysis. AI can automatically segment surgical video post hoc into key phases (induction, dissection, resection, anastomosis, closure, etc. This can aid in efficient review of surgeries, training (surgeons can get a summary of where time was spent in each phase), and even research correlating technique with outcomes. For instance, if an adverse event occurs, AI- analyzed video might pinpoint that a certain landmark was not identified or a wrong plane was entered, offering insights for quality improvement [64,65].

In summary, computer vision in HPB surgery is enabling a new level of intraoperative awareness. By recognizing anatomy, guiding the surgeon through critical steps (like confirming CVS), and flagging dangers, these AI systems act as an intelligent assistive layer over the surgical procedure. While still early-stage, evidence suggests such tools are feasible and can perform near expert-level in narrow tasks (e.g., identifying a specific landmark or bleeding source) [56,66,67,68]. They must, however, be rigorously tested for reliability, because false negatives or false positives could themselves pose risks (a missed alert or a false alarm could distract the team). Proper development and validation will be key. If successful, these systems could significantly enhance safety—one can envision essentially eliminating bile duct injuries or uncontrolled bleeding through earlier detection and standardized guidance [69]. Importantly, the surgeon remains in control at all times; the AI’s role is to support and enhance human performance, not replace it.

## 4. Discussion

**Figure 5:**
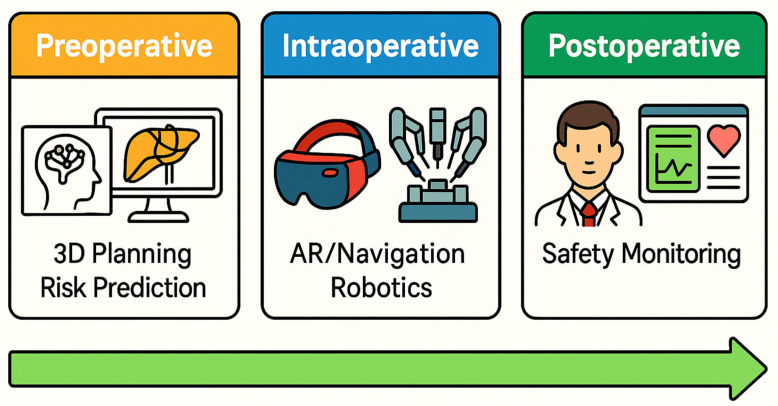
Conceptual overview of AI tools across the surgical timeline in HPB surgery. The figure summarizes representative tools and functionalities categorized by phase: preoperative (e.g., 3D reconstruction, surgical planning), intraoperative (e.g., augmented reality navigation, bleeding detection), and postoperative (e.g., outcome prediction, ICU forecasting). Colors denote stage—orange = preoperative, blue = intraoperative, green = postoperative. Green horizontal arrow indicates the continuum and progression of clinical workflow; it is directional only and does not imply magnitude, effect size or superiority. Icons are schematic as boundaries between stages may overlap in practice.

This systematic review reveals that the integration of AI and digital tools into HPB surgery is no longer theoretical—numerous proof-of-concept studies and early clinical evaluations have been conducted across the surgical pathway. Figure 5 provides a visual summary of the main categories of AI application during the preoperative, intraoperative, and postoperative stages Overall, AI applications show tremendous promise in augmenting surgeon capabilities, from improving diagnostic accuracy preoperatively to enhancing decision-making and precision intraoperatively. At the same time, our review highlights that most studies to date are preliminary, single-center investigations with relatively small sample sizes or short follow-up. Therefore, while the results are encouraging, broad claims of AI’s superiority should be tempered until larger-scale validation is achieved.

### 4.1. Key Findings and Current Evidence

AI systems show their strongest evidence in diagnostic imaging and risk prediction. Deep learning models can detect small liver lesions, predict recurrence, and identify pancreatic cancer with high accuracy, often matching or surpassing expert performance [70,71]. Machine-learning risk models outperform traditional scores for complications such as pancreatic fistula and liver failure, offering more individualized perioperative guidance [72,73].

In surgical planning, AI-driven 3D reconstructions improve anatomical understanding, with one RCT showing reduced operative time and blood loss. Intraoperatively, AR and computer-vision platforms enhance visualization and safety, recognizing landmarks like the critical view of safety, detecting bleeding in real time, and effectively improving the surgeon’s sensory and decision environment in real time [37,74].

However, most studies remain retrospective or pilot projects, with few randomized trials and limited patient outcome data. Evidence is strong for imaging accuracy but only preliminary for intraoperative applications, where sample sizes are small and heterogeneity is high.

### 4.2. Evidence Quality and Bias

Many included studies had methodological limitations. Common issues were small sample sizes, retrospective designs, single-center data, and lack of external validation—all of which raise the risk of bias. Using PROBAST [20], we found that a number of prediction model studies had an “unclear” or “high” risk of bias in the analysis domain, often due to lack of independent validation or non-transparent model building (e.g., some did not report how hyperparameters were optimized, raising concerns of overfitting). For diagnostic AI studies, patient selection bias was an issue—e.g., using only “ideal” images or excluding very challenging cases. There is also potential publication bias: successful applications are more likely reported than negative or neutral findings. We attempted to mitigate this by comprehensive searching and broad inclusion criteria, but it is possible that unsuccessful AI projects remained unpublished, skewing the literature toward positive results.

In terms of evidence grading, applying GRADE criteria is tricky for such heterogeneous literature. Imaging/diagnostics—moderate (consistent internal accuracy, limited external validation); predictive analytics—low-to-moderate; planning/3D and AR/MR—low; intraoperative computer vision—low.

Broadly, evidence for AI in imaging diagnosis could be considered moderate to high in certain niches (for instance, AI for detecting liver tumors has been reproduced in several settings, lending confidence to generalizability). On the other hand, evidence for AR and intraoperative AI is low to moderate—mostly limited to feasibility studies or small series without definitive proof of outcome benefit. No large trials or meta-analyses exist yet. This indicates we are at an early evidence level for intraoperative guidance tools.

Crucially, randomized controlled trials (RCTs) or other high-quality prospective studies are needed. We acknowledge RCTs are challenging in surgical technology research, but for AI, we may need creative trial designs, e.g., cluster-RCTs where certain centers adopt the AI tool and others use standard care or stepped-wedge designs as AI tools become available. Without such evidence, widespread adoption will lag, and clinicians will remain uncertain about true benefit. Encouragingly, some trials are underway—e.g., an international trial on AI-based coaching during cholecystectomy (using an AI to guide trainees) showed improved safety for trainees [75], though that pertains more to education than direct patient outcome. As more data emerge, future systematic reviews can better quantify effect sizes of AI interventions.

### 4.3. Safety, Ethical and Considerations

Integrating AI into surgery raises questions of responsibility, transparency, and equity. Surgeons remain accountable for decisions, but liability may blur if AI guidance proves misleading. Most current systems lack interpretability, underscoring the need for explainable AI and robust validation.

Data privacy and cybersecurity are critical as models rely on large surgical video and imaging datasets. Algorithmic bias is another risk: tools trained on narrow populations may underperform in diverse settings, widening disparities. Patient communication is essential—disclosure that AI or AR tools are used builds trust and respects autonomy.

Training and education must balance reliance on digital tools with fundamental surgical skills, due to a potential risk that future trainees become too dependent on navigation aids [76]. Regulatory oversight is still evolving, with agencies developing frameworks for AI as medical devices [77,78] and international guidelines emphasizing fairness, transparency, and accountability [79]. Proactive engagement with ethicists and regulators will be essential for safe adoption [80].

### 4.4. Costs and Implementation

AR/MR platforms, high-end workstations, and robotic integrations entail non-trivial acquisition and maintenance costs. Potential offsets include reduced operative time, lower blood loss, or fewer re-interventions when planning/guidance are effective; however, formal cost-effectiveness studies in HPB are scarce. Prospective evaluations with embedded economic endpoints are needed to inform adoption [81].

### 4.5. Clinical Guidance for Surgeons

From the current evidence, several practical lessons emerge:

- Preoperative planning: Use available 3D reconstructions and consider AI-based volumetry or segmentation where accessible, especially in complex resections.
- Intraoperative AR/Navigation: Treat AR overlays as adjuncts, not replacements, and verify with standard techniques. Early adoption should include fallback strategies.
- Robotic platforms: Maximize built-in digital features; integrate emerging AI modules cautiously and maintain oversight of semi-autonomous functions.
- AI decision support: Apply predictive models as aids, not determinants; combine outputs with clinical judgment and document their use.
- Team training: Ensure all OR staff understand the purpose and limits of digital tools, with contingency plans in place.
- Ongoing education: Surgeons should remain updated on AI principles and evolving guidelines to critically evaluate and responsibly deploy new technologies.
- Patient communication: Transparently explain when digital or AI tools support surgical planning or procedures and obtain informed consent for experimental systems.

By following these techniques, surgeons can responsibly embrace technology while maintaining high standards of care. The goal is to become “digitally augmented surgeons”—using all available tools to benefit patients but remaining the captain of the ship.

### 4.6. Strengths and Limitations of This Review

This review is one of the first comprehensive systematic reviews focusing specifically on AI in HPB surgery (as opposed to AI in surgery generally, or AI in radiology alone). By combining disparate study types under one umbrella, we provide HPB clinicians with a panoramic perspective of how AI might impact every step of the surgical process. A strength of our review is this breadth: contrary to current reviews that examine each aspect individually, readers can appreciate the continuum from preoperative AI diagnostics to intraoperative guidance and even postoperative analytics, all in the HPB context. We also included the most recent studies (through mid-2025), capturing very up-to-date advances such as AR-guided pancreatic surgery and AI-enhanced fluorescence imaging that were not covered in earlier reviews. We applied a robust multi-database search and used independent dual screening, which reduces the chance that we missed major relevant articles. Additionally, we assessed risk of bias and discuss evidence quality, adding a critical lens to interpret the findings responsibly rather than simply hyping technology.

On the flip side, by covering such breadth, our review sacrifices some depth in each subdomain. Each of the areas (e.g., radiomics for HCC, or AR for liver surgery) could warrant its own in-depth review or meta-analysis. We aimed for a balanced discussion relevant to clinical readers rather than data scientists, which means some technical details (like specific algorithm architectures or training techniques) are glossed over. Another limitation is the heterogeneity of the included studies, which precluded meta-analysis. We could not quantitatively synthesize effects, so our conclusions are qualitative and potentially subject to our interpretation bias. We also did not systematically examine cost-effectiveness—an important consideration for adopting these technologies. Currently, many tools are prototypes and cost data are scarce, but as tools mature, formal cost–benefit analyses will be needed (e.g., is an expensive AR system justified by a reduction in complications?).

Some limitations temper our findings such as 89% of studies were retrospective, risking selection bias, only 5/38 tools were externally validated, raising questions about scalability and ethical frameworks for AI-assisted surgery (e.g., informed consent protocols) remain undefined. Out of 38 studies, 62% had high risk of bias in analysis (PROBAST), primarily due to inadequate validation.

Furthermore, our search focused on the published literature; we might have missed very recent conference abstracts or industry research not in the public domain. However, by updating through mid-2025 and scanning references of relevant articles, we believe we captured the major developments. We also acknowledge that our review can become outdated quickly given the rapid evolution of AI—this is an inherent limitation when covering a fast-moving field. Our work is a “snapshot” up to mid-2025; readers should stay alert to new studies emerging literally every month.

Despite these limitations, we have tried to provide a comprehensive and up-to-date synthesis, with critical appraisal, to guide both surgeons interested in adopting new technologies and researchers aiming to identify gaps for future study.

## 5. Future Directions

The trajectory of AI in HPB surgery is clearly toward more integrated and intelligent systems. We anticipate that various AI components—imaging analysis, risk prediction, navigation, and vision assistance—will converge into unified platforms. For example, one can imagine an “HPB Surgery AI Suite” where a patient’s data are processed end-to-end: preoperatively, the system analyzes imaging and predicts potential difficulties; intraoperatively, it provides AR overlays and safety alerts; postoperatively, it analyzes surgical video to produce an operative report and even recommends improvements for the surgeon’s technique. Achieving this vision will require collaboration between surgeons, computer scientists, and industry engineers. Priority areas for robotic–AI integration include real-time instrument tracking, safety-critical alerts such as vessel proximity, and standardized API frameworks for interoperability. Integrating histopathology and molecular data with imaging/radiomics and clinical variables could also potentially improve prognostication and treatment selection; future multicenter datasets should explicitly include harmonized pathology inputs.

Data sharing and large datasets will be fundamental. As mentioned, many current models are limited by training data volume and diversity. Initiatives to create multi-institutional databases of annotated surgical videos (with proper anonymization and consent) are in progress and should be supported [82,83]. These will provide the “fuel” (big data) needed for robust algorithm development. Efforts like international surgical AI consortia or the establishment of shared video repositories can facilitate data sharing and standardize how surgical events are labeled.

Large Language Models (LLMs), such as ChatGPT-4, LLaMA, Mistral, and similar, represent another frontier that might intersect with HPB surgery. While LLMs are not directly performing surgical tasks, they could serve supporting roles in aggregating and delivering knowledge. For instance, an LLM-based assistant might be queried in real time for advice on an unusual intraoperative finding (“What are the management options if I encounter tumor thrombus in the portal vein?”), and it could rapidly provide synthesized knowledge from guidelines or literature. A recent editorial discussed how LLMs demonstrate potential in clinical documentation automation, complex data analysis, and real-time decision support in HPB surgery, but also highlighted challenges like AI “hallucinations” (confidently generating false information) and the need for oversight [84]. It is conceivable that future ORs might have voice-activated AI assistants—a surgeon could ask for recommended maneuvers or retrieve patient data hands-free. Before that happens reliably, LLMs need to become more accurate and context-aware, and hospitals would need to permit their use under secure conditions.

Another area is integrating precision medicine into surgical AI. As precision oncology advances, surgery will not remain an isolated discipline. We foresee AI enabling integration of genomic and molecular data into surgical decision-making. For example, if a patient’s tumor genomics suggest indolent disease, perhaps a limited resection or ablation could suffice versus an aggressive surgery. Conversely, high-risk biology might prompt a more extensive resection or multi-modal approach. Some groundwork for such “radiogenomic” models exists already [85]. In future HPB, one could imagine combining liver tumor genomics, imaging AI features, and anticipated regenerative capacity to tailor how much liver to resect for an optimal outcome. Though speculative, this aligns with the trend toward individualized therapy.

Usability and workflow integration: Future tools must be user-centric. One reason some technologies fail to gain traction is poor usability. Surgeons are busy and will not adopt something that significantly slows them down or is overly complex [86]. Future AR systems, for instance, should be near plug-and-play—automatically registering without lengthy manual calibration. The user interface should be intuitive, providing information in a minimally intrusive way (perhaps selectively showing critical info rather than cluttering the view). Haptic feedback or auditory alerts could complement visual AR to avoid information overload. The next generation of surgical robots might come with native AI integration, designed from the ground up with these features rather than tacked on after the fact. We expect upcoming robotic platforms (beyond da Vinci, such as newer systems by various companies) to advertise AI capabilities like automated sub-tasks, built-in AR imaging, and smart safety features (e.g., auto-pausing if an instrument nears a vital structure unexpectedly).

Validation and certification: Before routine clinical use, rigorous validation is needed. We anticipate more prospective trials in the next 5–10 years. Perhaps certain AI tools will undergo phase I/II style trials (assessing safety and feasibility, then efficacy). Regulatory bodies might stipulate that surgical AI meet specific performance benchmarks (for example, ≥95% sensitivity for critical structure detection). There may also be a role for simulation in validation—e.g., testing AI on recorded surgeries to see how often it would have prevented an adverse event or given false alarms. A kind of “digital twin” of clinical trials can be run on retrospective data to justify prospective testing. Post-market surveillance will also be crucial: AI systems might require periodic re-validation to ensure they perform as expected as data drift over time.

Education and training: The surgeon of the future needs to be adept with technology. Surgical residency programs may incorporate formal training on digital tools—for instance, sessions on how to interpret AI outputs, use AR goggles, or troubleshoot a robot’s AI feature. Just as surgical simulation is now part of training, we might see AI-assisted simulators where residents operate with an “AI mentor” that provides feedback. However, caution is warranted that reliance on AI does not diminish the development of fundamental surgical skills and judgment. Training curricula will have to emphasize AI as a support tool and ensure trainees can perform without it as well. Interestingly, AI might also personalize training—analyzing a trainee’s performance over cases and identifying specific areas to improve (e.g., speed of attaining CVS or economy of motion in suturing).

Interdisciplinary collaboration: We predict increasing collaboration between surgeons and data scientists. Some hospitals now have “clinical AI” teams that pair clinicians with AI developers to solve problems. In HPB surgery, surgeons should actively contribute to the development process: for example, by helping label data (what is important to identify in a video), defining clinical requirements (what accuracy would make a tool useful), and piloting prototypes. This co-development will ensure the final products truly meet clinical needs rather than being tech demos. The role of surgeon–data scientist is even emerging—clinicians who gain expertise in AI and drive projects from within.

Regulations and guidelines: The surgical community may develop its own guidelines for AI use, analogous to how societies issue guidelines on new technology adoption. We might see position statements on “Safe Use of Augmented Reality in Surgery” or “Standards for Reporting Surgical AI Studies” (some of which are already being discussed). As noted, the FUTURE-AI consortium has published principles for trustworthy AI in healthcare. Expect future guidelines to explicitly call for things like continuous monitoring of AI performance post-deployment and involving patients in decisions about AI use. Professional surgical bodies may also integrate AI topics into training curricula and certification (for example, mandates that surgeons demonstrate understanding of an AI tool before using it clinically).

In conclusion of future directions, the coming decade will likely transform how HPB surgery is planned and performed. We will move from the current phase of isolated pilot studies to integrated systems that assist the surgeon at each step. Realizing this future requires careful work on many fronts: technical innovation, clinical validation, ethical oversight, and surgeon education. If done correctly, HPB surgeons of the future will operate with greater knowledge (thanks to AI analysis), greater precision (thanks to real-time guidance), and greater confidence (thanks to predictive analytics and automation of tedious tasks). The focus must remain on patient outcomes—technology for its own sake is not the goal, but rather a means to safer, more effective, and more personalized surgical care.

## 6. Conclusions

AI across the HPB surgical pathway shows quite promising early results in risk prediction, planning, and intraoperative guidance. It is evident that before routine adoption, prospective, multicenter evaluations with external validation; standardized reporting; and economic assessment are needed as prerequisites. With continued refinement and careful implementation, these technologies could enhance precision, safety, and outcomes in HPB surgery.

## Figures and Tables

**Table 1:** Representative peer-reviewed studies on AI and digital tools in HPB surgery.

Study (Author, Year)	AI Method or Tool	Target Organ/Disease Focus	Clinical Application	Performance Metrics	Study Type
Bektas et al., 2024 [4]	Radiomics + CNN on ultrasound imaging	Liver (HCC tumors)	Imaging diagnosis (tumor classification; recurrence prediction)	AUC ~0.81–0.85 for early HCC recurrence (outperformed clinical risk factors); high accuracy distinguishing HCC vs. metastases	Retrospective cohort study (imaging analysis)
Tong et al., 2022 [22]	Deep CNN (ResNet-50) on EUS images	Pancreas (PDAC vs. chronic pancreatitis)	Imaging diagnosis (tumor differentiation)	AUC ≈ 0.95 for PDAC detection (vs. pancreatitis); demonstrated high diagnostic accuracy as decision support in borderline cases	Retrospective diagnostic study (multi-center image dataset)
Kawamura et al., 2023 [23]	CNN-based video analysis for critical view of safety (CVS) identification	Biliary (CVS during cholecystectomy)	Intraoperative safety guidance (anatomic landmark confirmation)	Real-time CVS recognition with ~83% overall accuracy; could alert surgeons to incomplete CVS, aiming to prevent bile duct injury	Retrospective observational study (analysis of recorded LC videos)
Müller et al., 2025 [24]	Ensemble ML algorithms on clinical data	Pancreas (postoperative fistula risk)	Outcome risk prediction (complication risk stratification)	AUC ~0.80 for clinically relevant POPF (vs. <0.60 with Fistula Risk Score); ML model significantly improved risk prediction	Retrospective cohort study (model development & validation)
Wang et al., 2025 [25]	Mixed reality (Hololens) with 3D liver model overlay	Liver (tumor cases)	Surgical planning (preoperative simulation & education)	Qualitative improvements in anatomical understanding and surgical strategy planning (holographic tumor visualization); no quantitative metrics reported	Prospective case series (pilot implementation of MR planning)
Giannone et al., 2021 [26]	Augmented reality overlay in robotic surgery	Liver (robotic hepatectomy for tumors)	Intraoperative guidance (real-time image guidance)	Feasible AR overlay of tumor and vasculature during surgery; improved lesion localization and transection planning (qualitative), though registration accuracy was a noted limitation	Prospective pilot study (feasibility in 3 cases)
Crisan et al., 2023 [27]	YOLOv5 deep learning vision + AR display	Intraoperative bleeding (multiple HPB procedures)	Intraoperative hazard detection (real-time bleed alert)	Detected surgical bleeding in real time with high accuracy (distinguished blood vs. irrigation fluid); demonstrated concept of automated hazard alerts via AR headset	Experimental feasibility study (lab validation on recorded surgery videos)
Pomohaci et al., 2025 [28]	N/A (Systematic review of AI in imaging)	Liver imaging (various pathologies)	Imaging diagnosis (lesion detection & classification)—review	N/A (Reviewed 329 studies: most focused on lesion classification; CT was the most common modality; highlighted lack of external validation in many studies)	Systematic review (2018–2024 literature)
Cheng et al., 2024 [29]	*Concept:* AI-enhanced fluorescence imaging	HPB cancers (tumor margins)	Intraoperative guidance (augmented fluorescence surgery)	N/A (Conceptual proposal: AI can enhance fluorescence image quality and differentiate tumor vs. normal tissue signals, potentially improving margin detection and resection precision)	Perspective article (conceptual review of emerging techniques)

Abbreviations: HCC—hepatocellular carcinoma; PDAC—pancreatic ductal adenocarcinoma; AUC—area under curve; CNN—convolutional neural network; ML—machine learning; LC—laparoscopic cholecystectomy; CVS—critical view of safety; AR—augmented reality; MR—mixed reality; POPF—postoperative pancreatic fistula.

## Data Availability

Every data extraction form and risk-of-bias table used in the upper manuscript are available from the authors upon reasonable request. No additional data or analytic code were generated.

## Supplementary Materials

The following supporting information can be downloaded at: https://www.mdpi.com/article/10.3390/jcm14186501/s1, Table S1: PRISMA 2020 Checklist; Table S2: Complete list of per-study details.

## Abbreviations

HCC—hepatocellular carcinoma; PDAC—pancreatic ductal adenocarcinoma; AUC— area under curve; CNN—convolutional neural network; ML—machine learning; LC—laparoscopic cholecystectomy; CVS—critical view of safety; AR—augmented reality; MR—mixed reality; POPF—postoperative pancreatic fistula; HPB—hepato-pancreato-biliary.
