# Supplementary material for: Artificial Intelligence and Digital Tools Across the Hepato-Pancreato-Biliary Surgical Pathway: A Systematic Review
Source: J Clin Med. 2025 Sep 15;14(18):6501. doi: 10.3390/jcm14186501 (PMC12470296; doi:10.3390/jcm14186501)
Supplement: Supplementary file 1 [file jcm-14-06501-s001.zip › Supplementary Table S2. Complete list of studies.pdf]

Supplementary_Table_S2__complete list of studies.

First Author (Year)	Country	Application Domain	Study Design	Dataset Size	Algorithm / Tool	Validation	Main Outcomes	Risk of Bias
**Lan (2025)**	China	Imaging & intra-op AR fusion (ablation)	Prospective feasibility/pilot	20 patients	Vision Pro AR image fusion	Internal only	Feasibility, usability	High
**Lyu (2024)**	China	Outcome prediction (POPF after PD)	Retrospective observational	322 patients	Deep learning classifier	Internal split	AUC 0.92 (pre/peri-op models)	Moderate
**Verma (2024)**	USA	Outcome prediction (POPF after PD)	Retrospective observational	500 patients	Machine learning (ensemble)	External validation	AUC 0.87	Moderate
**Ntourakis (2016)**	France	Intraoperative AR for liver metastases	Prospective feasibility/pilot	8 patients	AR overlay registration	None reported	Intra-op localization feasible	High
**Hua (2022)**	China	Intra-op bleeding detection (video)	Retrospective diagnostic	150 videos	Faster R-CNN	Internal split	Sensitivity ~0.89	Moderate
**Tong (2022)**	China	Imaging CEUS radiomics (PDAC vs CP)	Retrospective diagnostic	200 patients	DL radiomics	Internal	Accuracy ~0.88	Moderate
**Kawamura (2023)**	Japan	Intra-op CVS recognition (LC)	Prospective feasibility/pilot	54 videos	CNN classifier	Internal	Accuracy ~0.90	High
**Xi (2021)**	China	Imaging CT radiomics (HCC early recurrence)	Retrospective diagnostic	180 patients	Radiomics model	Internal	AUC ~0.82	Moderate
**Bereska (2024)**	Netherlands	Imaging CT resectability (pancreatic cancer)	Retrospective diagnostic	150 patients	AI assessment of vessel involvement	Internal	Accuracy ~0.85	Moderate
**Takamoto (2022)**	Japan	Pre-op 3D planning (virtual hepatectomy)	Retrospective observational	50 patients	AI liver reconstruction	Internal	Feasibility, planning accuracy	Moderate
**Kazami (2023)**	Japan	Pre-op 3D liver segmentation	Retrospective diagnostic	60 patients	Two-step AI segmentation	Internal	Automation rate, segmentation accuracy	Moderate
**Li L (2025)**	China	Pre-op AI-enhanced 3D printing vs standard	Randomized controlled trial	80 patients	AI-enhanced 3D printing workflow	External	↓ operative time, planning accuracy	Low
**Javaheri (2024)**	Germany	Wearable AR assistance (pancreas)	Prospective feasibility/pilot	10 patients	Head-mounted AR	Internal	Usability, workflow	High
**Wang J (2022)**	China	Outcome prediction (PHLF after hepatectomy)	Retrospective multicenter	900 patients	Machine learning	External validation	AUC ~0.85	Moderate
**Altaf (2025)**	Pakistan	Outcome prediction (bile leak post-hepatectomy)	Retrospective observational	400 patients	Machine learning	Internal	AUC ~0.81	Moderate
**Wang KD (2025)**	China	Imaging DCE-MRI radiomics (HCC recurrence)	Retrospective diagnostic	150 patients	Radiomics	Internal	AUC ~0.84	Moderate
**Oh (2024)**	South Korea	AR visualization in 3D liver laparoscopy	Prospective feasibility/pilot	20 patients	AR overlay	Internal	Usability, registration error	High
**Ramalhinho (2023)**	United Kingdom	AR value/usability in liver laparoscopy	Prospective feasibility/pilot	15 surgeons (simulated/clinical)	AR overlay	Internal	Usability metrics	High
**Wu XW (2023)**	China	AR-assisted navigation for PD	Retrospective observational	40 patients	AR navigation system	Internal	↓ blood loss, time (short-term)	Moderate
**Cheng K (2022)**	China	Phase recognition (LC)	Retrospective diagnostic	200 videos	Deep learning	Internal	Accuracy ~0.86	Moderate
**Tokuyasu (2021)**	Japan	Anatomical landmark detection (LC)	Retrospective diagnostic	80 videos	Deep learning	Internal	Accuracy ~0.82	Moderate
**You (2024)**	China	Phase recognition (PD)	Retrospective diagnostic	150 videos	Deep learning	Internal	Accuracy ~0.85	Moderate
**Laplante (2023)**	Canada	Safe chole AI platform validation	Prospective feasibility/pilot	30 videos	AI platform	Internal	Accuracy ~0.87	High
**Golany (2022)**	Israel	Phase recognition (complex LC)	Retrospective diagnostic	120 videos	Deep learning	Internal	Accuracy ~0.84	Moderate
**Yang (2024)**	South Korea	Phase recognition (LC)	Retrospective diagnostic	110 videos	Deep learning	Internal	Accuracy ~0.83	Moderate
**Pei (2025)**	China	Bleeding detection (surgical video)	Retrospective diagnostic	200 videos	Deep learning detector	Internal	Sensitivity ~0.88	Moderate
**Romero (2019)**	Canada	Imaging CT liver lesion classification	Retrospective diagnostic	250 images	Deep CNN	Internal	Accuracy ~0.85	Moderate
**Huo (2020)**	USA	Imaging CT liver lesion characterization	Retrospective diagnostic	300 images	Deep dynamic texture learning	Internal	AUC ~0.88	Moderate
**Wang D (2025)**	China	POPF prediction after laparoscopic PD	Retrospective observational	180 patients	Machine learning	Internal	AUC ~0.80	Moderate
**Wang P (2025)**	China	Pre-op 3D recon + intra-op AR fluorescence	Retrospective observational	50 patients	3D + AR fluorescence guidance	Internal	↓ operative time / EBL	Moderate
**Wu S (2024)**	China	AI-based chole coaching (training)	Randomized controlled trial	100 trainees	AI coaching platform	External	Improved skill score	Low
**Banerjee (2015)**	USA	CT radiogenomics biomarker (HCC)	Retrospective diagnostic	120 patients	Radiogenomics	Internal	MVI prediction; outcome assoc.	Moderate
**Stanuch (2021)**	Poland	Mixed reality visualization (GI incl. HPB)	Prospective feasibility/pilot	10 patients	MR overlay (HoloLens 2)	Internal	Feasibility/usability	High
**Crisan (2023)**	Romania	Hazard detection in robotic oncologic surgery	Retrospective diagnostic	80 videos	Deep learning detector	Internal	Accuracy ~0.86	Moderate
**Morales-Galicia (2025)**	Mexico	Outcome prediction (cholangiocarcinoma)	Retrospective observational	Not reported	Machine learning	Internal	OS/DFS prediction metrics	Moderate
**Zureikat (2016)**	USA	Robotic vs open PD (digital platform)	Retrospective observational	Not reported (multi-institutional)	Robotic platform (no AI)	None	Perioperative outcomes	Moderate
**Woo (2014)**	South Korea	TilePro multi-image display (robotic)	Prospective feasibility/pilot	Not reported	Digital display integration	None	Workflow feasibility	High
**Agrawal (2025)**	India	3D imaging & virtual surgical planning	Retrospective observational	Not reported	3D VSP workflow (AI-assisted)	Internal	Complications, planning precision	Moderate
